# Individual Calculation of Effective Dose and Risk of Malignancy Based on Monte Carlo Simulations after Whole Body Computed Tomography

**DOI:** 10.1038/s41598-020-66366-2

**Published:** 2020-06-11

**Authors:** Markus Kopp, Tobias Loewe, Wolfgang Wuest, Michael Brand, Matthias Wetzl, Wolfram Nitsch, Daniela Schmidt, Michael Beck, Bernhard Schmidt, Michael Uder, Matthias May

**Affiliations:** 1 Department of Radiology, University Hospital Erlangen, Erlangen, Germany; 2 Institute of Medical Microbiology and Hygiene, University of Technology Dresden, Dresden, Germany; 3 Department of Nuclear Medicine, University Hospital Erlangen, Erlangen, Germany; 4 Siemens Healthineers GmbH, Forchheim, Germany

**Keywords:** Cancer, Tomography, Whole body imaging, Risk factors

## Abstract

Detailed knowledge about radiation exposure is crucial for radiology professionals. The conventional calculation of effective dose (ED) for computed tomography (CT) is based on dose length product (DLP) and population-based conversion factors (k). This is often imprecise and unable to consider individual patient characteristics. We sought to provide more precise and individual radiation exposure calculation using image based Monte Carlo simulations (MC) in a heterogeneous patient collective and to compare it to phantom based MC provided from the National Cancer Institute (NCI) as academic reference. Dose distributions were simulated for 22 patients after whole-body CT during Positron Emission Tomography-CT. Based on MC we calculated individual Lifetime Attributable Risk (LAR) and Excess Relative Risk (ERR) of cancer mortality. ED_MC_ was compared to ED_DLP_ and ED_NCI_. ED_DLP_ (13.2 ± 4.5 mSv) was higher compared to ED_NCI_ (9.8 ± 2.1 mSv) and ED_MC_ (11.6 ± 1.5 mSv). Relative individual differences were up to −48% for ED_MC_ and −44% for ED_NCI_ compared to ED_DLP_. Matching pair analysis illustrates that young age and gender are affecting LAR and ERR significantly. Because of these uncertainties in radiation dose assessment automated individual dose and risk estimation would be desirable for dose monitoring in the future.

## Introduction

The Euratom council directive (*2013/59*/*Euratom*) emphasizes the need for patient radiation dose monitoring in clinical routine^1^. Computed tomography (CT) is indispensable for contemporary patient care, but many studies suggest a relation between low dose protracted radiation exposure and an increased incidence of malignancy based on the linear Non-threshold Dose-Response Model^2^. Therefore, detailed knowledge of radiation exposure from CT examinations is crucial for radiology healthcare professionals in clinical routine to detect and address increased risks of malignancy. Widely used parameters for radiation exposure assessment like the volumetric CT dose index (CTDI_vol_) and the Dose Length Product (DLP) characterize scanner radiation output, but are unable to take individual patient characteristics into account^3^. Conversion to effective dose (ED_DLP_) is feasible using population-based conversion factors (k) that take the averaged radiosensitivity in defined anatomic regions into account^4^. A variety of different k-factors are recommended in the literature for different volumes (e.g. head, thorax, abdomen, pelvis) and different CT-scanners^5,6^. These k-factors are mostly derived from phantom models that try to represent average patient anatomy in western populations, but are unable to respect individual anatomy like missing organs due to aplasia or resection, organ hypo- or hypertrophy, skeletal deformations and metal implants. Nevertheless, these programs, like e.g. the National Cancer Institute (NCI) dosimetry system for CT, can be considered as current academic reference (ED_NCI_)^7^. However, the routinely performed calculation of ED_DLP_ is imprecise and the degree of difference to the real ED in each individual patient is unknown. Risk estimates that are calculated on these imprecise data could then easily be misinterpreted. Therefore, conventionally calculated ED_DLP_ can be used for comparison between different CT-scanners or different examination protocols, but should not be used for comparison between different patient collectives or for radiation risk estimation^8,9^.

Stochastic estimation of radiation exposure from CT is feasible by Monte Carlo (MC) simulations. Romanyukha *et al*. used computational phantoms and MC-simulations to calculate body size-specific conversion factors from regression curves^10^. Huda *et al*. were able to show that this phantom based technique is also feasible to derive organ doses and to use these for cancer risk estimation^11^. Only few studies with small numbers of mostly pediatric patients used MC simulations for individual dose and risk estimation per patient, probably due to the high computational power needed^12,13^. The main drawback of MC calculations is that they are limited to the reconstructed body volume. Therefore, overexposure in z-axis and scattered radiation to the organs outside the directly exposed volume cannot be taken into account.

Detailed information about biological effects of low-level ionizing radiation is available from the National Academy of Sciences report number seven about *Health Risks from Exposure to Low Levels of Ionizing Radiation* (BEIR VII). The authors of BEIR VII report follow the linear Non-threshold Dose-Response Model and presume that the risk of cancer incidence and mortality due to medical examinations are mainly based on low-level ionizing radiation dose, age and gender^14^.

The aim of this study was to provide individual calculations of ED in whole-body exposure derived from MC calculations (ED_MC_) and to compare them with the conventional method (ED_DLP_) and the academic reference (ED_NCI_) following an equivalence hypothesis in a heterogeneous study collective. Furthermore, cancer risk estimates that are based on ED_MC_ should allow to evaluate the impact of patient characteristics on individual risk of malignancy. Whole body CT from combined Positron Emission Tomography (PET)-CT examinations were exclusively selected in order to minimize the indeterminate radiation dose to body parts outside the imaging volume that cannot be assessed by MC.

## Methods

### Study design

**Figure 1:**
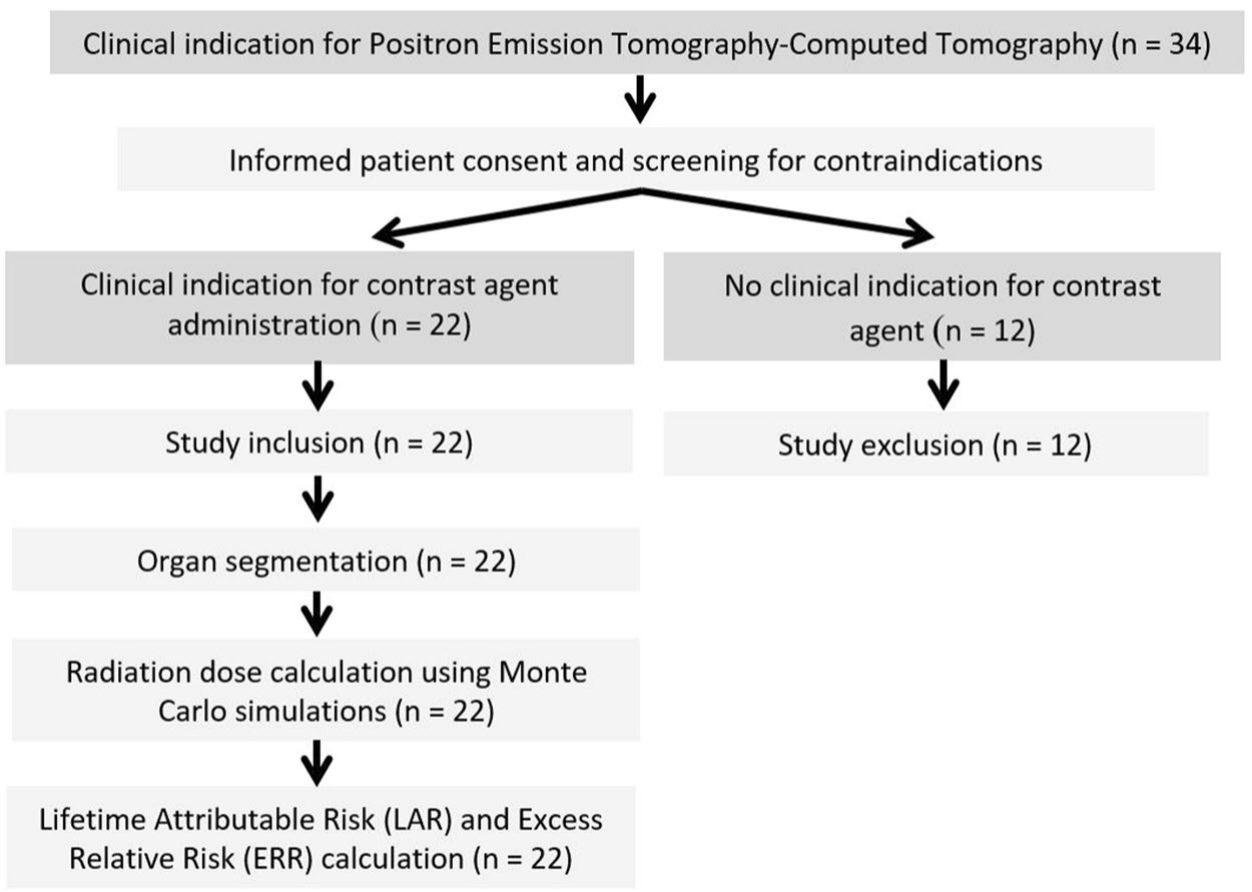
Illustration of study design.

Twenty-two patients from a collective of 34 consecutive patients, with clinical indication for whole body PET-CT, were retrospectively included (Fig. 1). Inclusion criteria were the complete and artifact-free coverage of the scan volume, sufficient intra-venous contrast injection and full availability of individual patient data including age, height, weight and body mass index (BMI). Exclusion criteria were severe artifacts (n = 0) and examinations without contrast injection (n = 12). Clinical indications were infectious diseases (n = 8), bronchial carcinoma (n = 6), head and neck cancer (n = 3), cancer of unknown primary (n = 2), malignant melanoma (n = 2) and retroperitoneal fibrosis (n = 1).

Written informed consent was obtained and archived for each patient. The study was approved by the local ethics committee of the Friedrich-Alexander University Erlangen-Nuremberg and complied with the Declaration of Helsinki.

### Image acquisition

All examinations were performed with continuous volume acquisition using a Siemens Biograph True Point 64 PET-CT (Siemens Healthcare GmbH, Forchheim, Germany). Indication, supervision and image analysis were performed by senior physicians with extensive experience in PET-CT (>6 years). CT was performed from head to mid-femur by a single spiral acquisition in a cranio-caudal direction using a tube voltage of 120 kV, anatomic tube current modulation with a reference tube-current time product of 170 mAs, pitch 0.8, gantry rotation time 0.5 seconds and 64 × 0.6 mm slice acquisition by 32 × 0.6 mm detector collimation and z-axis flying focal spot double sampling^15^. Unlike in diagnostic CT, where different body parts (e.g. head/neck and thorax/abdomen) are often examined in multiple contrast phases and different positions, PET-CT uses single spiral acquisitions with only one single bolus injection allowing for complete coverage of all body organs without overlapping of anatomic regions. Therefore, the effect of scattered radiation to all radiosensitive tissues can be taken into account by MC calculations and the effect of overranging in the beginning and end of the spiral acquisition can be neglected^16^. All patients received body weight adapted intravenous injection of ^18^F-fluorodeoxyglucose (3 MBq/kg). After an average resting period of 91 (±25.6) minutes iodine containing contrast agent (Ultravist 370^®^, Bayer-Schering Healthcare, Berlin, Germany) was mechanically administered into an antebrachial cannula using a power injector (Accutron CT-D, Medtron AG, Saarbruecken, Germany), and the single spiral CT acquisition was started with a fixed delay of 70 s. The PET acquisitions were run subsequently in 7–8 steps, depending on patient length and exam volumes. For this study full field of view (500 × 500 mm) images were reconstructed using a smooth filtered back projection kernel (B31), slice thickness 5.0 mm and increment 5.0 mm. The resulting voxel size was 0.98 × 0.98 × 5.00 mm.

### Conventional calculation of effective dose

Tube current time product, CTDIvol and DLP were recorded for each examination from the PET-CT scan protocol as provided by the scanner. Details about their definition and calculation are reported elsewhere in literature^6^. The main drawback of these monitoring techniques is that they are unable to provide information about the biological impact of radiation exposure and should only be taken as an index of radiation output by the CT system for comparative purposes^9,17^. The biologically relevant effective dose (ED_DLP_) was calculated for each examination by multiplication of DLP and the region-specific conversion factor. For this study k_Body_ (0.015 mSv/mGy∙cm) was used as recommended by the American Association of Physicists in Medicine^6^. Dose exposition related to 18F-fluorodeoxyglucose (FDG)-PET were not considered for ED calculation.

### Phantom based calculation of effective dose

Academic reference ED was calculated using a dedicated CT dosimetry software tool provided by the National Cancer Institute (NCI-CT)^7^, which combines reference phantoms provided by the International Commission on Radiological Protection (ICRP) and MC simulations of a reference CT scanner (Somatom Sensation 16, Siemens Healthcare GmbH, Forchheim, Germany). Individual examination parameters, gender, age, height and body weight were used as input for this mathematical phantom based calculation (ED_NCI_). Dedicated descriptions about computational methods of this software are available in the literature^7^.

### Organ segmentation

**Figure 2:**
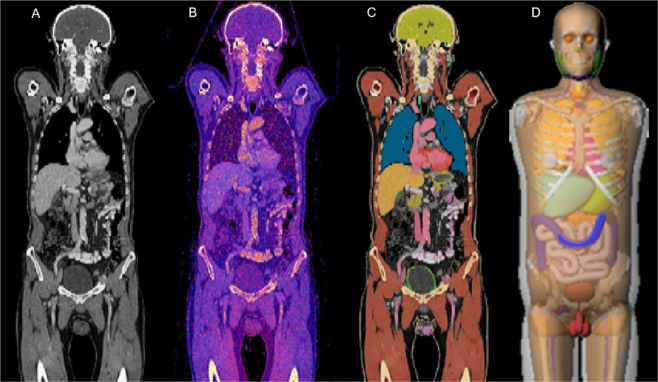
Calculation of effective dose: (**A**) contrast-enhanced CT acquisition; (**B**) relative dose distribution from MC simulations; (**C**) organ segmentation used for organ dose calculation; (**D**) phantom illustration of the National Cancer Institute dosimetry system for CT.

All radiosensitive organs or tissues including all remainders, which are mentioned in IRCP report 103 were segmented using a dedicated software package (ITK-SNAP 1.8, Penn Image Computing and Science Laboratory, Philadelphia, USA). Semi-automatic threshold segmentation with manual corrections was used for the lungs, cortical bone, bone marrow, liver, spleen, brain, heart, muscle and skin. All other organs or tissues were segmented manually in a slice per slice fashion. Therefore, missing organs due to aplasia or resection, organ hypo- or hypertrophy, skeletal deformations and metal implants were considered. For hollow organs, only voxels contributing to the wall were considered to contain radiosensitive tissues. Segmentation of lymphatic tissues was restricted to visible lymphatic nodes (Fig. 2). All segmentations were performed by a specially trained radiology resident (4 years experience) and reviewed from an experienced board-certified radiologist (9 years experience).

### Monte Carlo based calculation of effective dose

**Figure 3:**
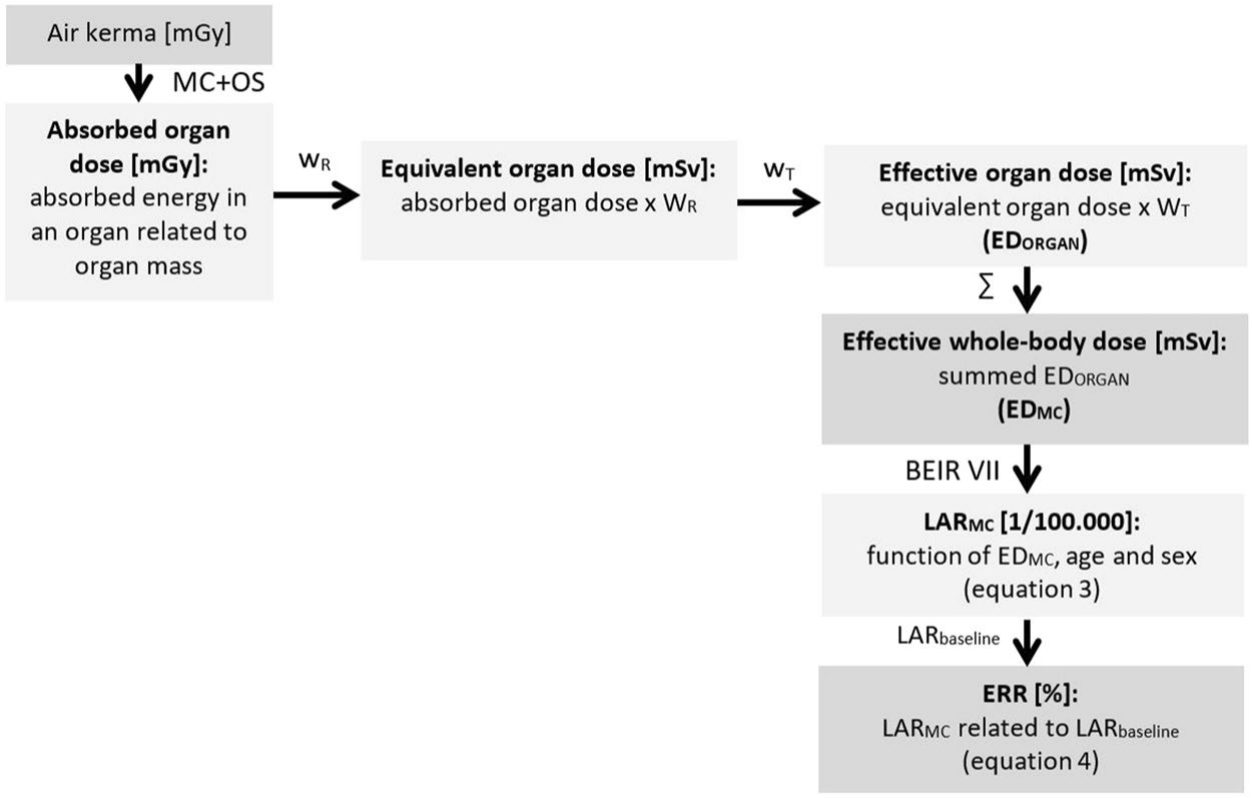
Flow chart illustrating radiation dose terms in computed tomography (CT): Air karma, Monte Carlo simulations (MC) and organ segmentations (OS) are used for absorbed organ dose calculation. Multiplication of absorbed organ dose with radiation weighting factors (W_R_) results in equivalent organ dose. For calculation of effective organ dose (ED_ORGAN_) tissue weighting factors (W_T_) are required. Effective whole-body dose (ED_MC_) is the sum (∑) of each ED_ORGAN_. Data from BEIR VII report was used to estimate Lifetime Attributable Risk (LAR_MC_) of cancer mortality and to calculate its relation to the baseline of lifetime attributable cancer risk as Excess Relative Risk (ERR).

Extensive mathematical models in simulation techniques can be utilized to calculate dose distribution for each voxel of CT scan volumes using the attenuation values and geometry from DICOM datasets as input^18,19^. All MC dose simulations were carried out using the software package ImpactMC (VAMP GmbH, Erlangen, Germany). Software details and information concerning the Monte Carlo calculation algorithms are reported elsewhere^18,20^. Multiplication of the resulting relative dose values per voxel with air kerma provides the absorbed dose for each voxel. The air kerma is a scanner specific value, which describes the kinetic energy transferred in air dependent on geometry and tube settings. It can be considered as measurement of x-ray beam intensity without object, which was 18.3 mGy for this study. The averaged absorbed dose over an organ volume, defined from segmentations, provides the absorbed organ dose. Equivalent organ dose was calculated by multiplication with the radiation weighting factor for photons (W_R_ = 1) and used for subsequent effective organ dose calculations (ED_Organ_) by multiplication with the dedicated tissue weighting factor (W_T_) as recommended in ICRP report 103^21^. The sum of all effective organ doses provides ED_MC_ for each individual (Fig. 3). The relative contribution of the respective ED_Organ_ to the ED_MC_ was calculated following Eq. 1:

1 $${{\rm{C}}}_{{\rm{Organ}}}={{\rm{ED}}}_{{\rm{Organ}}}/{{\rm{ED}}}_{{\rm{MC}}}$$

Individual conversion factors (k_MC_) were calculated using Eq. 2 to illustrate the differences compared to the conventional calculation method using DLP and k_body_.

2 $${{\rm{k}}}_{{\rm{MC}}}={{\rm{ED}}}_{{\rm{MC}}}/{\rm{DLP}}$$

### Radiation risk assessment

Lifetime Attributable Risk (LAR) is defined as risk of disease of an exposed cohort in comparison to a non-exposed cohort. Calculations of LAR for cancer mortality were based on report VII about Biologic Effects of Ionizing Radiation (BEIR VII). The BEIRV VII report refers to an individual radiation dose exposure of 100 mGy. Assuming a linear risk distribution between decennium age intervals LAR_MC_ was derived from ED_MC_ by linear interpolation between the younger (N_y_) and older (N_O_) cohort as shown in equation 3 using patient age (A_p_) and the age of the younger cohort (A_y_) as input values^22^.

Equation 3: Calculation of individual LAR

$$LA{R}_{MC}=\left({N}_{y}-\left(({N}_{y}-{{\rm{N}}}_{0})\ast \frac{{A}_{p}-{A}_{Y}}{10\,{\rm{years}}}\right)\right)\ast \frac{E{D}_{Organ}}{100\,{\rm{mGy}}}$$

The Excess Relative Risk (ERR_MC_), as a measurement of the exceeding risk of an exposed person compared to a non-exposed person, was calculated using the solid cancer mortality in the United States as baseline (female: 17500/100000; male: 22100/100000) using equation 4^23^.

Equation 4: Calculation of individual ERR

$$ER{R}_{MC}=LA{R}_{MC}/LA{R}_{baseline}$$

### Pairwise patient comparison

Because of the rather small patient collective in this study, mainly due to the extensive effort needed for segmentation of all radiosensitive tissues, pairwise patient comparisons were selected to highlight the influence of individual patient characteristics on the radiation dose parameters and radiation risk estimation. Matching patient pairs, each with two comparable values and one variable parameter, were found for the parameters age, sex and BMI.

### Statistical analysis

All statistical analyses were performed using the software package SPSS Statistics Version 21 (IBM, Somers, NY, USA). Normal distribution of the data was tested by Kolmogorov-Smirnov and Shapiro-Wilk test. Normally distributed data is presented as mean ± standard deviation. Median and range are provided if no normal distribution was assumed. Illustration is provided as Bland-Altman plots. Spearman’s rank order test was used to test for correlations between tube current, BMI, ED_DLP_ and ED_MC_. The significance level was defined as p < 0.05.

## Results

### Demographics of the study collective

Five out of 22 patients (23%) were female and 17 male (77%). The mean age was 57.3 ± 14.3 years and the mean BMI was 26.0 ± 5.9 kg/m². Three patients (13.6%) were younger than 40 years. The female patient group was younger (53.6 ± 17.6 years) compared to the male patient group (58.4 ± 13.6 years). Female patients had a lower mean BMI (21.2 ± 2.5 kg/m²) compared to male patients (27.4 ± 5.88 kg/m^2^). From the male subgroup 9 patients (52.9%) suffered from overweight (BMI > 25), and three patients (17.6%) had severe overweight (BMI > 30). One female and one male patient were considered as underweight (BMI < 19). No overweight patient was found in the female subgroup. Detailed patient characteristics are shown in Table 1.

**Table 1:** Demographic characteristics and body mass index (BMI) of the patient collective.

BMI [kg/m^2^]		<20	20–25		>25	>30	total
**Age [years]**							
<40		1♂	1♀	1♂			n = 3
40–55		1♀	1♀	4♂	1♂		n = 7
55–70			1♀	2♂	3♂	1♂	n = 7
>70			1♀		1♂	3♂	n = 5
	total	n = 2	n = 11		n = 5	n = 4	n = 22

### Organ specific radiation dose exposure

Mean equivalent organ dose was 13.0 ± 3.5 mGy. Highest equivalent organ dose values were found in high attenuating structures such as the cortical bone and the thyroid gland. Lowest values were found in profound organs like the extra-thoracic (ET) respiratory region and the uterus. Many superficial organs like muscles (11.5 ± 2.0 mGy), breast tissue (10.9 ± 0.8 mGy) and salivary glands (14.3 ± 2.8 mGy) also had rather low equivalent organ dose values. Despite its profound position well perfused kidneys had very high equivalent organ dose values (17.0 ± 2.1 mGy).

**Figure 4:**
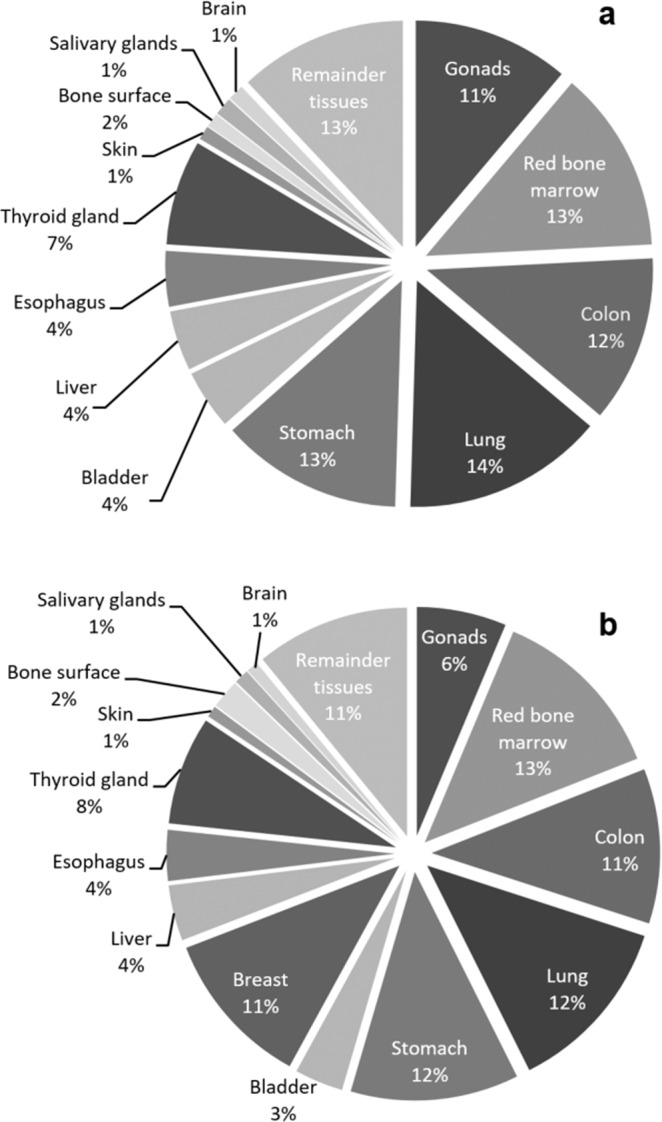
Effective organ dose distribution for male (**a**) and female (**b**) patient subgroup.

Effective organ dose was mainly influenced by W_T_, nicely demonstrated by the thyroid gland. It has the second highest mean equivalent organ dose (21.2 ± 5.4 mGy), but is only ranked 7^th^ highest effective organ dose (8% of total organ dose distribution) because of the rather low W_T_ (0.04). In contrast, the lungs ranked only 7^th^ in equivalent organ dose (13.4 ± 1.9 mGy), but received highest effective organ dose levels in female and male patients (12% and 14% of the total ED_MC_) due to high W_T_ (0.12). Colon, lungs and stomach contributed to more than 50% of the total ED_MC_ in male patients and to 48% in female patients (Fig. 4). Differences between W_T_ and the relative contribution of the effective organ dose to whole body ED_MC_ (C_Organ_) reflect the influence of individual radiation dose distribution on each organ dose. Highest positive differences between W_T_ and C_Organ_ were found for the bone surface (♀: +102.1%; ♂: +134.8%), the thyroid gland (♀: +89.1%; ♂: +78.1%) and the kidneys (♀: + 49.4%; ♂: +47.1%). The male gonads also had substantially higher C_Organ_ than expected from its rather low W_T_ ( + 38.2%), but the female gonads had the highest negative difference of all organs (−21.8%). Other organs with high negative differences were the adrenal glands (♀: −18.3%; ♂: −11.8%). Detailed organ dose information is shown in Table 2 and organ-based LAR of cancer mortality is provided for all organs listed in BEIR VII. Considering the similar effective organ doses of the lung (1.48 ± 0.15 mSv) and the breast (1.30 ± 0.09 mSv) the LAR for pulmonary malignant disease (13.25 ± 4.24/100.000) was remarkably higher compared to the LAR for breast cancer mortality (2.46 ± 1.62/100.000).

**Table 2:** Illustration of equivalent organ dose, tissue weighting factors (w_T_), effective organ dose (ED_ORGAN_), the relative contribution of ED_ORGAN_ to whole-body effective dose (C_ORGAN_) and estimation of organ-specific Lifetime Attributable Risk (LAR) of cancer mortality based on Monte Carlo simulations and report VII about Biologic Effects of Ionizing Radiation (BEIR VII).

Organs	Equivalent organ dose [mGy]	W_T_	Effective organ dose (female) [mSv]	Effective organ dose (male) [mSv]	C_ORGAN_ (female)	C_ORGAN_ (male)	LAR of cancer mortality [n/100000]	
Red bone marrow	12.3 (±2.3)	0.12	1.49 (±0.24)	1.47 (±0.28)	0.125	0.126		
Colon	11.2 (±2.8)	0.12	1.28 (±0.08)	1.36 (±0.38)	0.108	0.116	4.69 (±1.88)	
Lung	13.4 (±1.9)	0.12	1.48 (±0.15)	1.64 (±0.38)	0.125	0.14	13.25 (±4.24)	
Stomach	12.2 (±1.5)	0.12	1.39 (±0.06)	1.48 (±0.23)	0.117	0.127	1.43 (±0.47)	
Breast	10.9 (±0.8)	0.12	1.30 (±0.09)		0.11		2.46 (±1.62)	
Gonads	14.6 (±4.1)	0.08	0.74 (±0.07)	1.29 (±0.26)	0.063	0.111	1.94 (±0.84)	
Bladder	11.7 (±2.0)	0.04	0.40 (±0.05)	0.49 (±0.06)	0.034	0.042	1.88 (±0.47)	
Liver	12.0 (±1.2)	0.04	0.46 (±0.04)	0.49 (±0.08)	0.039	0.042	1.22 (±0.41)	
Esophagus	11.1 (±1.4)	0.04	0.42 (±0.05)	0.45 (±0.01)	0.035	0.039		
Thyroid gland	21.2 (±3.2)	0.04	0.90 (±0.04)	0.83 (±0.05)	0.076	0.071		
Skin	11.0 (±2.1)	0.01	0.09 (±0.00)	0.11 (±0.03)	0.08	0.01		
Bone surface	26.6 (±4.7)	0.01	0.24 (±0.01)	0.27 (±0.03)	0.02	0.023		
Salivary glands	14.3 (±2.8)	0.01	0.12 (±0.01)	0.15 (±0.03)	0.01	0.013		
Brain	12.7 (±3.3)	0.01	0.10 (±0.00)	0.14 (±0.03)	0.08	0.012		
Remainder tissues								
Spleen	12.4 (±1.7)	0.0092	0.11 (±0.01)	0.12 (±0.02)	0.009	0.01		
Kidney	17.0 (±2.1)	0.0092	0.16 (±0.01)	0.15 (±0.02)	0.014	0.013		
Heart	12.5 (±1.5)	0.0092	0.12 (±0.01)	0.12 (±0.01)	0.01	0.01		
Pancreas	11.7 (±1.2)	0.0092	0.11 (±0.01)	0.11 (±0.01)	0.009	0.009		
Oral mucosa	13.5 (±3.1)	0.0092	0.12 (±0.01)	0,13 (±0.03)	0.009	0.011		
Lymph nodes	12.6 (±2.2)	0.0092	0.12 (±0.02)	0.12 (±0.02)	0.009	0.01		
Muscle	11.5 (±1.9)	0.0092	0.11 (±0.01)	0.11 (±0.02)	0.008	0.009		
Small intestine	13.2 (±1.9)	0.0092	0.12 (±0.00)	0.13 (±0.02)	0.009	0.011		
Gall bladder	11.6 (±1.3)	0.0092	0.11 (±0.01)	0.11 (±0.01)	0.008	0.009		
Adrenal gland	10.2 (±1.3)	0.0092	0.09 (±0.01)	0.10 (±0.01)	0.008	0.008		
Prostate	11.4 (±3.2)	0.0092		0.10 (±0.03)		0.008	0.74 (±0.25)	
Uterus	9.9 (±1.1)	0.0092	0.09 (±0.01)		0.008		0.28 (±0.09)	
Extrathoracic respiratory (ET) region	8.7 (±7.1)	0.0092	0.08 (±0.06)	0.07 (±0.07)	0.009	0.006		

### Effective dose and individual radiation risk assessment

**Figure 5:**
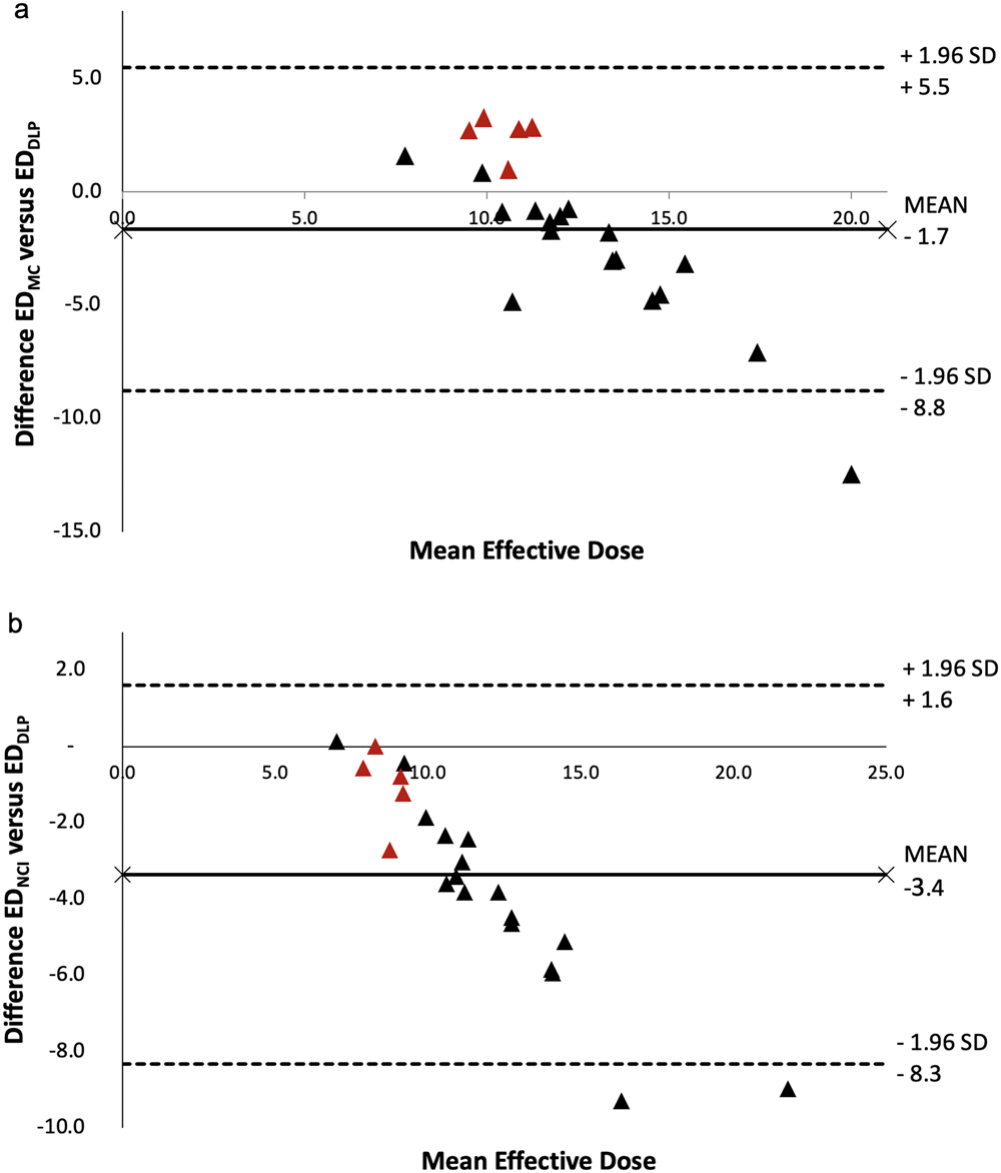
(**a**) Bland-Altman plot for the difference between conventional effective dose calculation (ED_DLP_) and calculation based on Monte Carlo simulations (ED_MC_): The difference between both methods increases with high and low values of mean ED. This implicates that in high and low mean ED the conventional ED_DLP_ over- and underestimates radiation dose exposure compared to ED_MC_. (**b**) Bland-Altman plot for the difference between ED_DLP_ and calculation by the National Cancer Institute dosimetry system for CT (ED_NCI_): The difference between both methods increases especially with high values of mean ED. This suggests that in high mean ED the conventional ED_DLP_ overestimates radiation dose exposure compared to ED_NCI_ (red triangles indicate female patients).

**Figure 6:**
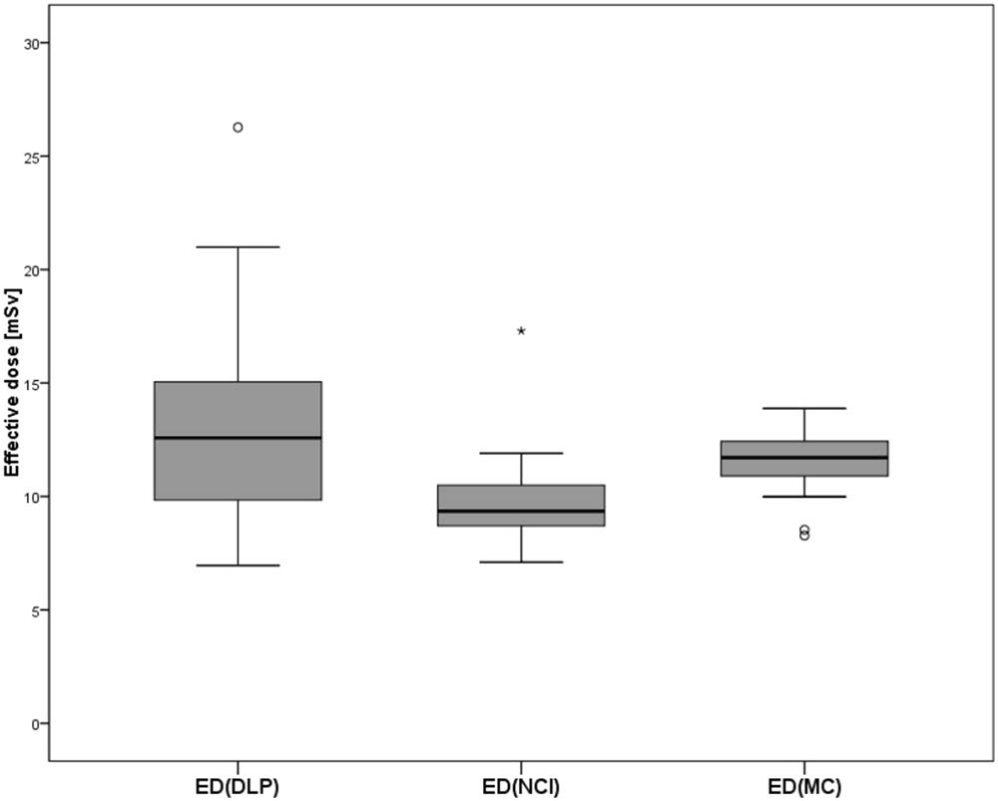
Comparison of effective dose for ED_DLP_, ED_NCI_ and ED_MC_ illustrated as boxplot.

ED_DLP_ (13.2 ± 4.52 mSv) was higher than ED_MC_ (11.6 ± 1.47 mSv) and ED_NCI_ (9.8 ± 2.1 mSv). Particularly high differences between ED_MC_ and ED_DLP_ were found for patients with relatively high or low radiation dose exposure. Mean difference between both methods was −1.7 mSv (Fig. 5a). The same tendency was found for the comparison of ED_NCI_ and ED_DLP_ but with higher mean negative differences −3.4 mSv (Fig. 5b). A comparison between the three calculation methods is illustrated as boxplot (Fig. 6). The range of radiation dose was substantially smaller in both advanced methods (ED_MC_: 5.6 mSv, ED_NCI_: 10.2 mSv) compared to the conventional technique (ED_DLP_: 19.3 mSv). Effective mAs and DLP linearly increased with higher BMI in Spearman’s rank order test (r_s_ = 0.961 and 0.949). This effect should be mainly due to the anatomy-based tube current modulation algorithm. Therefore, also BMI and ED_DLP_ had a high correlation coefficient (r_s_ = 0.949). The correlation between ED_MC_ and BMI (r_s_ = 0.644) was less and differences between ED_DLP_ and the advanced techniques was especially high in over- and underweight patients. For example, the highest ED_DLP_ of 26.3 mSv was found in a 77-year-old man suffering from adiposity grade 3 (BMI 40.0 kg/m²) while ED_NCI_ (17.3 mSv) and ED_MC_ (13.9 mSv) were substantially lower (34% and 47% less). Lowest ED_DLP_ of 7.0 mSv was calculated for a 30-year-old underweight man (BMI 16.8 kg/m²), which was close to ED_NCI_ (7.1 mSv), but substantially lower than ED_MC_ (8.5 mSv, 21% higher). Consequently, the individually calculated conversion factors (k_MC_) have a range from 53% to 140% when referred to k_Body_ from literature. The mean k_MC_ (0.014 ± 0.004 mSv/mGy cm) approximately reflects the established value (0.015 mSv/mGy cm), which therefore seems to be suitable for regular weight patients. However, only one fourth of the patients in this study (n = 6, 27.2%) had less than 10% difference between k_MC_ (0.0135–0.0165 mSv/mGy cm) and k_Body_ (mean BMI 22.95 ± 1.58, range 20.7–25.0 kg/m²).

The values of LAR were especially high in young female patients. The highest value (60/100.000) was calculated for a 30-year-old, normal weight woman (BMI 21.6 kg/m²). In contrast, low LAR values were calculated for older, predominantly male patients. The lowest value (23/100.000) was found for a 76-year-old, overweight man (BMI 29.8 kg/m²). Extent of ED_MC_, ED_DLP_ and LAR often differed considerably when compared between young and old or underweight and obese patients, while the interaction between these individual parameters was rather difficult to predict. Detailed results of radiation dose and risk calculations are provided in Table 3.

**Table 3:** Individual patient and radiation exposure characteristics with illustration of dose length product (DLP), volume computed tomography dose index (CTDIvol), effective mAs (eff. mAs), effective dose based on conventional calculation method (ED_DLP_), Monte Carlo simulations (ED_MC_) and the dosimetry system for CT provided by the National Cancer Institute (ED_NCI_). ED_NCI_/ED_DLP_ and ED_MC_/ED_DLP_ deviations, conversions factors (k_MC_), Lifetime Attributable Risk (LAR_MC_) and Excess Relative Risk (ERR_MC_) are individually calculated.

age [y]	sex	weight [kg]	BMI	DLP [mGy · cm]	CTDIvol [mGy]	eff. mAs [mAs]	ED_DLP_ [mSv]	ED_NCI-CT_ [mSv]	ED_MC_ [mSv]	ED_NCI_/ED_DLP_ deviation [%]	ED_MC_/ED_DLP_ deviation [%]	k_MC_	LAR_MC_*	ERR_MC_ [%]
30	W	70	21.6	675	5.8	86	10.1	7.4	11.1	−26.7	9.9	0.016	60	0.34%
48	W	54	21.1	545	5.4	80	8.2	7.6	10.9	−7.3	32.9	0.020	52	0.30%
55	W	46	17.3	554	5.5	81	8.3	8.3	11.5	0.0	38.9	0.021	51	0.29%
56	W	62	21.5	634	6.3	93	9.5	8.7	12.3	−8.4	29.5	0.019	53	0.30%
79	W	65	24.5	656	6.5	96	9.8	8.6	12.7	−12.2	29.6	0.019	26	0.15%
30	M	51	16.8	464	4.6	68	7.0	7.1	8.5	1.4	21.4	0.018	33	0.15%
36	M	68	24.1	783	6.8	100	11.8	9.4	10.9	−20.3	−7.6	0.014	41	0.19%
44	M	80	25.0	841	7.3	107	12.6	9.2	11.9	−27.0	−5.6	0.014	44	0.20%
52	M	84	25.9	827	7.1	105	12.4	8.8	11.1	−29.0	−10.5	0.013	39	0.18%
53	M	77	24.3	841	7.3	107	12.6	9.6	10.9	−23.8	−13.5	0.013	38	0.17%
53	M	72	24.3	836	7.2	106	12.5	10.1	11.5	−19.2	−8.0	0.014	40	0.18%
54	M	67	20.7	630	6.4	92	9.5	9.0	10.3	−5.3	8.4	0.016	35	0.16%
57	M	125	37.3	1399	12.1	178	21.0	11.7	13.9	−44.3	−33.8	0.010	46	0.21%
58	M	84	25.9	875	7.6	111	13.1	9.3	8.3	−29.0	−36.6	0.001	27	0.12%
59	M	78	24.6	949	8.2	121	14.2	10.4	12.4	−26.8	−12.7	0.013	40	0.18%
61	M	90	29.4	1135	9.8	145	17.0	11.9	13.9	−30.0	−18.2	0.012	43	0.20%
67	M	67	23.0	724	6.3	92	10.9	9.0	10.0	−17.4	−8.3	0.014	27	0.12%
69	M	86	28.7	1003	8.65	128	15.1	10.4	12.1	−31.1	−19.8	0.012	31	0.14%
73	M	102	33.7	1135	9.79	145	17.0	11.1	12.5	−34.7	−26.5	0.011	28	0.12%
74	M	100	31.9	1130	9.75	144	17.0	11.1	12.1	−34.7	−28.8	0.011	26	0.12%
76	M	86	29.8	998	8.61	127	15.0	10.5	11.9	−30.0	−20.7	0.012	23	0.10%
77	M	105	40.0	1751	17.36	165	26.3	17.3	13.8	−34.2	−47.5	0.008	25	0.11%
57.3 (±14.3)	♀ = 22.7%	78.1 (±19.0)	26.0 (±5.9)	881.1 (±301.0)	7.9 (±2.8)	112.6 (±28.6)	13.2 (±4.5)	9.8 (±2.1)	11.6 (±1.5)	−22.3 (±12.0)	−5.8 (±23.6)	0.014 (±0.004)	37.6 (±10.6)	0.18 (±0.07)

*Lifetime attributable risk (LAR) for all cancer mortality/100.000 persons.

### Pairwise patient comparison

**Figure 7:**
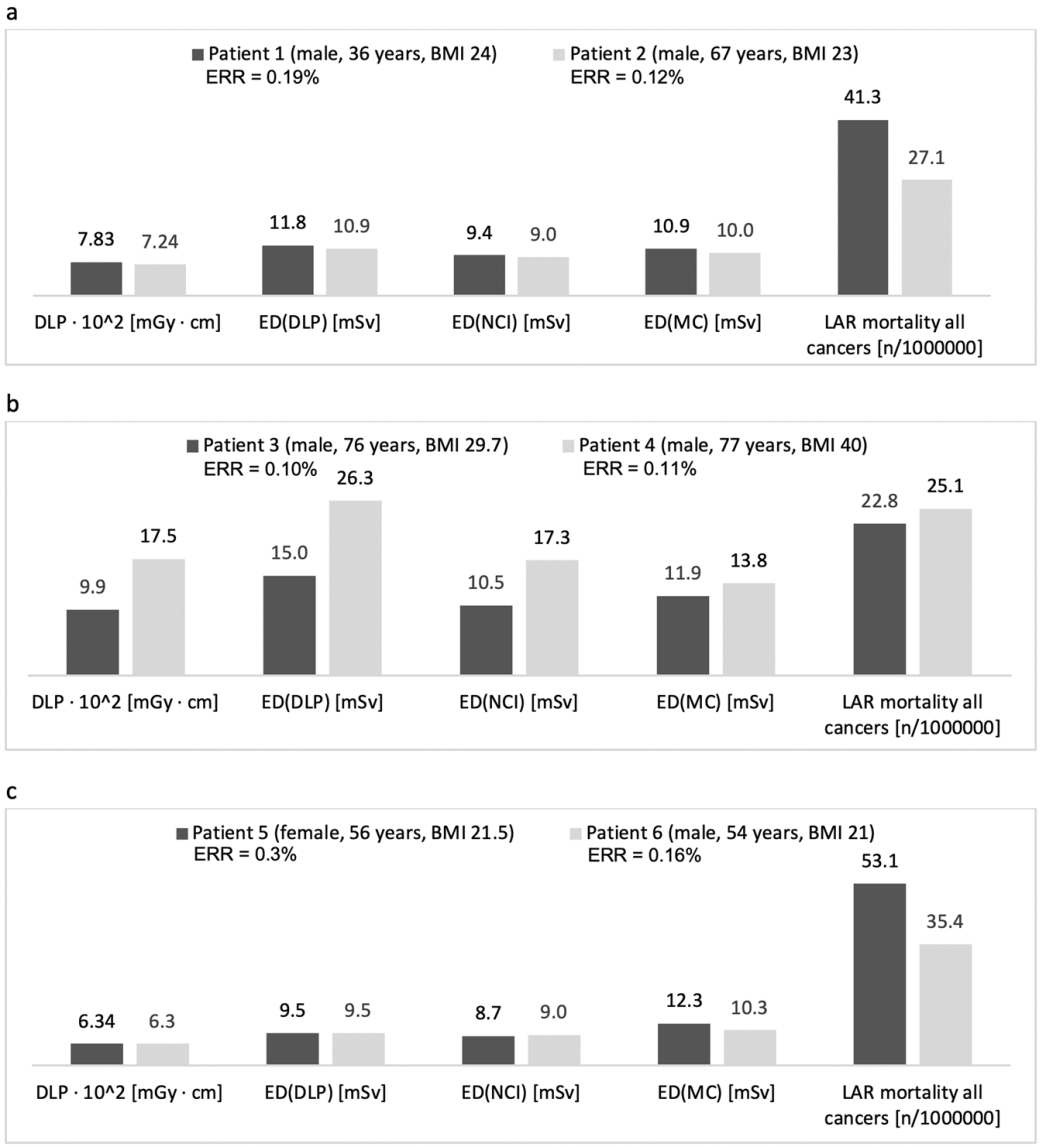
Pairwise comparison of dose length product (DLP), effective dose (ED), Lifetime Attributable Risk (LAR) and Excess Relative Risk (ERR) in dependence of age (**a**), Body Mass Index **(b**) and sex (**c**).

Two male patients with matching constitution (BMI 24 vs. 23 kg/m²) but different age (36 versus 67 years) had comparable DLP (783 vs. 724 mGy · cm, −7.5%), ED_DLP_ (11.8 vs. 10.9 mSv, −7.5%), ED_NCI_ (9.4 vs. 9.0 mSv, −4.3%) and ED_MC_ (10.9 vs 10.0 mSv, −8.4%). Nevertheless, calculations for LAR of cancer mortality (41.3 vs. 27.1/100.000; −34.4%) and ERR (0.19 vs. 0.12%, −36.8%) differed considerably. The young age seems to be the decisive factor among all the considered factors, accounting for about 50% higher risk estimates (Fig. 7a).

Two male patients with matching age (76 and 77 years) but different BMI (29.7 vs. 40.0 kg/m², +34.7%) were exposed to considerably different DLP (998 vs. 1751 mGy·cm, +75.4%) based on anatomic tube current modulation. Therefore, ED_DLP_ calculations provided very high values for the obese patient (15.0 vs. 26.3 mSv, +75.5%). Dose differences calculated with ED_NCI_ (10.5 vs. 17.3 mSv, +64.5%) were also considerably high, while ED_MC_ values ware nearly comparable (11.9 vs. 13.8 mSv; +15.9%). Differences in LAR_MC_ (22.8 vs. 25.1/100.000; +9.6%) and ERR (0.10 vs 0.11%; +10.0%) were even less (Fig. 7b). BMI seems to have only a minor influence on effective dose and risk estimation, while conventional methods would have resulted in an overestimation of almost 100%.

Two patients with matching age (56 and 54 years) and BMI (21.5 vs 21.0 kg/m²) but different sex had similar DLP (634 vs. 630 mGy·cm; +0.63%). ED was the same using the conventional method in both patients (ED_DLP_ = 9.5 mSv) and almost the same with the NCI method (ED_NCI_ = 8.7 vs 9.0 mSv; −3.3%). ED_MC_ calculation was slightly higher for the female patient (12.3 vs. 10.3 mSv; +19.4%), but LAR (53.1 vs. 35.4/100000; +50.2%) and ERR (0.3% vs. 0.16%; +87.5%) were substantially higher. Sex seems to have a dominant impact on the risk estimation. Moreover, the unfavorable combination of age and sex leads to the highest values in the entire study collective, despite average ED values (Fig. 7c).

## Discussion

Individual radiation dose assessment and risk calculation is feasible by image based Monte Carlo simulations and organ segmentations in an adult clinical routine collective that underwent full body exposure in a single spiral acquisition. This is in good agreement with the findings of Li *et al*. and Tian *et al*. who evaluated comparable techniques for radiation dose estimation in small pediatric collectives (n = 2 and n = 42)^16,24^. A comparable approach for cancer risk estimation in adults based on anthropomorphic phantoms was described by Huda *et al*.^11^. In contrast to these prior studies, we provide a combination of individual radiation dose analysis, voxel-based organ segmentation and cancer risk estimation in direct comparison to such phantom based estimation and the DLP method. The need for such an advanced dose monitoring method, due to several uncertainties with the conventional techniques, has been raised elsewhere in literature^20,25^.

In our study high equivalent organ doses were strongly related to radiodensity (e.g. bone surface and high contrast medium uptake in the kidneys, the thyroid gland and the extra-thoracic respiratory region), which seems to be a much stronger predictor for equivalent organ dose than anatomical organ position. This is in good agreement with the contrast media related increase of DNA double-strand break foci after radiation exposure reported in a prior study for CT^26^.

W_T_ is the strongest predictor of effective organ dose. For example, the thyroid gland and the kidneys have the second and third highest equivalent organ dose (21.2 ± 3.2 mSv and 17.0 ± 2.1 mSv). However, due to low tissue weighting factors (W_T_ = 0.04 and 0.0092) effective organ dose of the thyroid gland and the kidneys were only sixth and twelfth highest.

Our results confirm that individual patient characteristics have a considerable impact on radiation dose calculation, which is underrepresented by the conventional method. The calculated error in our adult collective (−48 to 39%) is comparable to the previously published study results for children (−63 to 28%)^19^. Individually calculated k_MC_ from our study reveals that the routinely used k_body_ from literature can be applied to larger clinical collectives, but are limited in their value for individual risk assessment^6^. The DLP-method slightly overestimates the effective dose in general, but it seems to be appropriate in regular weight male patients. However, especially in female and underweight patients, underestimation of the effective dose may be critical for risk perception in the clinical setting. Moreover, its overestimation in obese patients may lead to restrained use of high exposure parameters, and then to poor or insufficient image quality. Individual anatomic characteristics (e.g. missing organs due to aplasia or resection, organ hypo- or hypertrophy, skeletal deformations, metal implants) may lead to considerable changes of radiation dose distribution and consequently of individual risk. Even phantom based estimation methods, like ED_NCI_ in this study, are unable to take these individual properties into account^7^. Bland-Altman analysis demonstrated that these phantom based estimates have the tendency to underestimate ED in general, while the effect of only moderate increase of ED in obese patients despite the very high energy exposure is confirmed.

The influence of personal risk profiles on cancer risk estimates are not yet represented in standard radiation dose reporting systems. It can further increase the error of perceived and true risk of radiation dose from CT examinations. The combination of individual dose distributions and risk assessment has been presented for a few other examinations in literature, such as absorptiometry and spine radiographs, but not for spiral CT examinations^27^.

Pairwise patient comparisons provide a comprehensive overview of the extent of error with regard to age, BMI and sex. Especially younger patients are prone to higher LAR and ERR since more active cell division and longer life expectancy after radiation exposure is presumed^28^. In our example the ERR of a patient in his mid-thirties was 1.6-fold the ERR of a patient in his mid-sixties and the ERR for a female patient in her mid-fifties was almost doubled compared to a matching male patient. The increasing tube current by anatomy-based modulation in obese patients seems to be of less importance to the patients’ cancer risk. CTDI and ED_DLP_ were 1.8-fold higher for a high-grade obese patient compared to an overweight patient with matching age and sex, while the ERR was only 10% higher. Therefore, future dose assessment strategies should not only focus on absolute dose values. Furthermore, reporting LAR or ERR seems to be much more appropriate in clinical routine and a more comprehensible value for the patient-physician interaction.

Some limitations have to be considered while interpreting this study. First, the study population consisted of a heterogeneous, small and retrospectively selected patient collective with an imbalanced ratio of female to male patients. The small number of patients is mainly due to the high computing power required for MC calculations and the work intense manual organ segmentation that we used for this study. We estimate that the increasing computing power and automated organ segmentations by the application of artificial intelligence could overcome this limitation in the near future^29,30^. Second, only full body dose exposure is reported in this study. This avoids indeterminate scattered radiation to radiosensitive structures and over-ranging effects, but limits the findings to this clinically rather rare indication. Further evaluations for limited examination volumes could become feasible with retrospective simulations from these data in larger collectives by future studies. Third, all examinations were conducted on the same scanner. The findings of this study can therefore not automatically be transferred to other CT systems^5,24^. Forth, all tissue weighting factors that are provided in literature so far are averaged for sex and age, which may probably limit their applicability for patient specific risk estimation. Fifth, the linear Non-threshold Dose-Response Model itself is discussed controversially among experts for diagnostic dose levels^31,32^. Sixth, LAR and ERR calculations in this study are only related to low dose radiation exposure. They illustrate the individual radiation dose related risk to develop cancer. Obviously, the overall individual cancer risk for a primary or even a secondary cancer and also the life expectancy is potentially much more influenced by age, genetic make-up, efficiency of DNA damage repair, therapy-related adverse effects and many other influencing factors.

The importance of patient specific dose surveillance is illustrated by this study. ED_DLP_ can be used for radiation dose assessments in larger collectives, but individual considerations require advanced techniques like ED_MC_. Conventional methods tend to underestimate radiation dose in underweight and female patients. Therefore, these patients have to be evaluated with special care. The influence of young patient age and female gender on cancer risk estimates is very high. Thus, radiation dose assessment should not only provide whole body or organ dose measurements but also individual risk calculations, which could be included in future surveillance programs.

## Data Availability

The datasets generated during and analyzed during this study are available from the corresponding author on reasonable request.
